# Identification and Defensive Characterization of *PmCYP720B11v2* from *Pinus massoniana*

**DOI:** 10.3390/ijms23126640

**Published:** 2022-06-14

**Authors:** Bin Liu, Yini Xie, Huanhuan Yin, Zhichun Zhou, Qinghua Liu

**Affiliations:** 1 Research Institute of Subtropical Forestry, Chinese Academy of Forestry, Hangzhou 311400, China; lb_binbin@163.com (B.L.); xieyini0430@163.com (Y.X.); 2 Zhejiang Provincial Key Laboratory of Tree Breeding, Hangzhou 311400, China; 3 Zhengzhou Botanical Garden, Zhengzhou 450007, China; yhh183762710@163.com

**Keywords:** *Pinus massoniana*, pine wood nematode, *PmCYP720B11v2*, tissue-specific expression, defense response, drought stress tolerance

## Abstract

*Pinus massoniana* is a pioneer species for afforestation timber and oleoresin, while epidemics of pinewood nematode (PWN; *Bursaphelenchus xylophilus*) are causing a serious biotic disaster for *P. massoniana* in China. Importantly, resistant *P. massoniana* could leak copious oleoresin terpenoids to build particular defense fronts for survival when attacked by PWN. However, the defense mechanisms regulating this process remain unknown. Here, *PmCYP720B11v2*, a cytochrome P450 monooxygenase gene, was first identified and functionally characterized from resistant *P. massoniana* following PWN inoculation. The tissue-specific expression pattern and localization of *PmCYP720B11v2* at the transcript and protein levels in resistant *P. massoniana* indicated that its upregulation in the stem supported its involvement in the metabolic processes of diterpene biosynthesis as a positive part of the defense against PWN attack. Furthermore, overexpression of *PmCYP720B11v2* may enhance the growth and development of plants. In addition, *PmCYP720B11v2* activated the metabolic flux of antioxidases and stress-responsive proteins under drought conditions and improved drought stress tolerance. Our results provide new insights into the favorable role of *PmCYP720B11v2* in diterpene defense mechanisms in response to PWN attack in resistant *P. massoniana* and provide a novel metabolic engineering scenario to reform the stress tolerance potential of tobacco.

## 1. Introduction

Pine wilt disease (PWD), a catastrophic forest pandemic driven by the pinewood nematode (PWN; *Bursaphelenchus xylophilus*), which is disseminated by *Monochamus* beetles [1] (Kamata et al., 2013), can cause major economic and ecological devastation wherever it is spread [2,3]. It is extremely damaging to conifers, especially to *Pinus massoniana* Lamb, causing high mortality and infection rates of the hosts. China is currently the country most endangered by the PWN. The epidemic had expanded to 18 provinces in China, encompassing 650,000 ha, by 2017 [2,3]. it has reportedly destroyed approximately 5,000,000 m^3^ of *P. massoniana* since the first detection of PWN in Nanjing, China, in 1982 [4].

*P. massoniana*, serving as a pioneer species for afforestation timber and oleoresin, is the most common and widespread native conifer in China. It is widely used in construction, medicine, and papermaking [5]. Unfortunately, *P. massoniana* is a susceptible host of PWN, facing the heavy threat of PWD. Importantly, some natural individuals of *P. massoniana* appear to survive outbreaks of PWD in any given place [6], implying that some natural mutant genotypes show PWN resistance. Understanding the resistance mechanisms of such resistant genotypes may be a critical and effective approach to addressing this issue.

Terpenoids play a critical role in plants’ ability to adapt to biotic stress [7,8]. As the primary chemical defenses of conifers, oleoresin terpenoids are comprised of numerous mixtures of over 100 different monoterpenes (C10), sesquiterpenes (C15), and diterpenes (C20), mainly in the form of nonvolatile diterpene resin acids (DRAs) [7,9,10]. These terpenoid combinations may protect conifers from pests and infections such as bark beetles, weevils, and the fungus that they carry [11,12,13]. Herbivores and diseases cause substantial oleoresin buildup in the resin ducts of all major plant organs when they attack conifer tissues [13]. Additionally, conifers have a dynamic terpenoid defense strategy, and the qualitative content of the diverse terpenoids in oleoresin can be altered dramatically in response to biotic stress [14,15,16].

Cytochrome P450 monooxygenases (CYP450s) are the driving force contributing to the diversity of the terpenes produced by terpene synthases [17,18]. In plants, the great majority of terpene oxidations have been identified thus far, and they are catalyzed by P450s in stereospecific places and manners. These oxidations can occur consecutively at a specific site to form carboxylic acids, or they can result in rearrangements of the terpene backbone structure, such as the formation of new C-C bonds. Additionally, certain enzymes are capable of oxidizing a given backbone in various locations or similar substrates in vitro or in other species. In the final step of the reactions, the oxidized terpenoids are further functionalized by the addition of acyl, benzoyl, glycosyl, or even alkaloids [19].

CYP450 proteins are the largest protein superfamily, and they have long been known for their wide range of biocatalytic functions. They are extensively distributed in higher plants and have roles in both primary and secondary metabolite production. Above all, CYP 450 enzymes are ubiquitous in the secondary metabolite biosynthetic pathways involved in the production of defense chemicals to biotic stress, especially for long-lived sessile coniferous trees. In addition, they are also believed to improve the adaptation of plants to abiotic stressors [20].

Drought, as one of the major abiotic stressors, has adversely influenced the growth and development of plants [21]. For better and longer survival, plants have evolved sophisticated mechanisms to detect and respond to the harsh and changeable environment [22,23], such as physiological and or molecular processes where they regulate the concentrations of diverse phytohormones or the expression of stress-response genes [24,25,26]. *CYP450s* are critical contributors in the evolutionary adaptation strategies, and multiple *CYP450s* have been identified [25]. For instance, a *CYP450*, *OsDSS1*, was known to be involved in growth and drought stress responses in *Oryza sativa* L. (Tamiru et al., 2015). *Gh_D07G1197* and *Gh_A13G2057*, belonging to CYP450s, play putative roles in drought and salt stress tolerance in *Gossypium hirsutum* [27]. *SoCYP85A1* from *Spinacia oleracea* L. was involved in root development and drought stress in transgenic tobacco [28]. However, the identification and function of *CYP450s* in conifers, especially in *P. massoniana,* are insufficient.

The expansion of CYP450s in land plants has built a large family in different plant metabolisms. The CYP720B group is a conifer-specific member of the CYP450 family, whereas it has been reported in only a few conifer species thus far. *PtCYP720B1* was the first *CYP720B* gene identified in *Pinus taeda*, which was proven to be a multifunctional and multisubstrate cytochrome CYP450 monooxygenase. It could catalyze two consecutive oxidations of abietadienol and abietadienal to form abietic acid. In addition, it converted dehydroabietadiene and isopimaradiene to their alcohol and aldehyde forms. Then, it was also a catalyst to turn levopimaradienol into levopimaric acid [29].

In different spruce and pine genomes and transcriptomes, 15 different *CYP720B* genes were found [30]. Eight different genes were functionally characterized from the conifer-specific CYP720B subfamily in white spruce. In addition, CYP720B4 from Sitka spruce was reported to produce dehydroabietic acid [31], which is linked to weevil resistance [32]. The combination of coniferous diterpene synthase and CYP720B-P450 exemplifies the modularity of diterpene metabolism in rosin biosynthesis [33]. The variable combination of di-TPS and CYP720B synthase produces a series of defensive metabolites, such as diterpene alcohols, aldehydes, and DRA, in this metabolic system. Given that CYP720B-catalyzed oxidations contribute to defensive metabolites, it is critical to functionally identify the CYP720Bs implicated in the terpenoid biosynthesis pathway.

However, the function of CYP720B in *P. massoniana* remains uncertain. *PmCYP720B11v2*, which was differentially expressed in a recent transcriptome screen of resistant *P. massoniana* following PWN inoculation [34], was first functionally described in the current study. The expression pattern and functional characterization of *PmCYP720B11v2* supported the hypothesis that its expression may play a favorable role in the metabolic processes connected to the terpene defense in response to a PWN attack. Furthermore, the overexpression of *PmCYP720B11v2* in transgenic tobacco (*Nicotiana benthamiana*) boosted the growth and development of the plants and contributed to improving their stress tolerance.

## 2. Results

### 2.1. Cloning and Sequence Analysis of PmCYP720B11v2 from P. massoniana

To functionally characterize the possible involvement of *PmCYP720B11v2* in the PWN-induced defense in *P. massoniana*, *PmCYP720B11v2*, a significantly differentially expressed gene induced by PWN inoculation [34], was cloned based on a full-length transcriptome of *P. massoniana*. The full-length CDS of *PmCYP720B11v2* was 1461 bp, encoding 486 amino acids with 20 conserved motifs (Figures S1 and S2).

**Figure 1:**
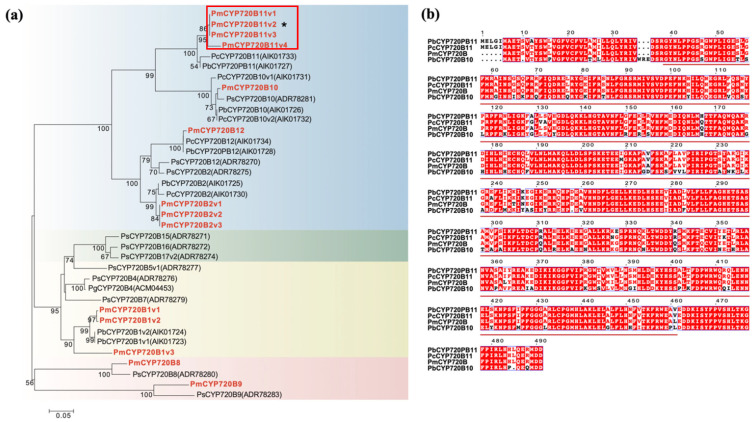
Phylogenetic analysis of CYP720Bs from *P. massoniana*. (**a**) Phylogenetic analysis of CYP720B enzymes from *P. massoniana*, *P. contorta*, *P. banksiana*, *Picea sitchensis*, and *P. glauca*. The maximum-likelihood tree was obtained using the JTT method by CIPRES. Bootstrap values are shown as a percentage of 1000 replicates. The red fonts indicate PmCYP720Bs from *P. massoniana*. Asterisks (*) indicate the enzyme that was characterized in this study (PmCYP720B11v2). (**b**) Amino acid alignments of PmCYP720B11v2 from *P. massoniana*.

To further characterize the sequence of *PmCYP720B11v2*, a phylogenetic tree was built to determine the evolution of *PmCYP720B11v2* among *P. massoniana* and other species (Figure 1a). The CYP720Bs of gymnosperms can be divided into four clades (I-IV), and it can be seen from the PmCYP720B evolutionary tree that 14 PmCYP720Bs of *P. massoniana* can be divided into four clades from I-IV. *PmCYP720B11v2* was located in clade I of the *CYP720B* family, most closely related to *PcCYP720B* from *P. contorta*, up to approximately 100%.

A multisequence alignment analysis of the PmCYP720B11v2 amino acid sequence of *P. massoniana* using Clustal X showed that the *PmCYP720B11v2* amino acid sequence has a typical conserved domain (Figure 1b). The C-terminal binding region with hemoglobin, containing a conserved F-G-R-C-G-G domain, is the main feature of CYP450 proteins. The N-terminal has a transmembrane signal with a hydrophobic sequence and contains a conserved region of proline. Even though the CYP450 protein has a long evolutionary relationship, *CYP720B* in higher plants has homology.

### 2.2. Tissue-Specific Expression Patterns and Localization of the PmCYP720B11v2 Gene in Resistant P. massoniana

**Figure 2:**
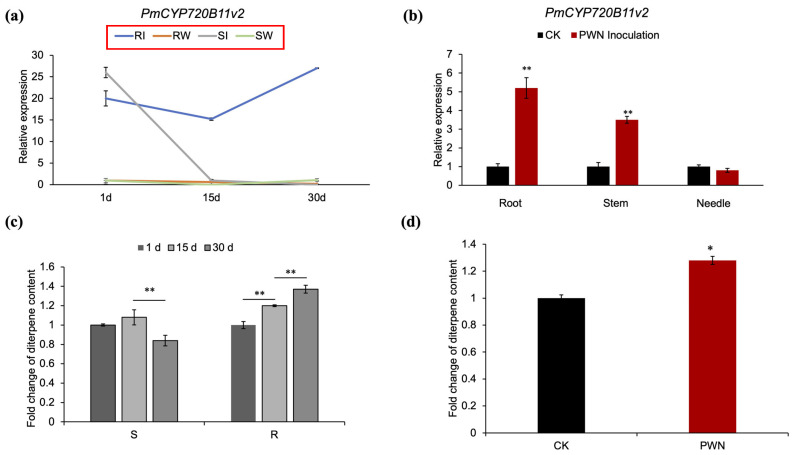
Tissue-specific expression patterns of *PmCYP720B11v2* and analysis of diterpene changes in *P. massoniana* following PWN inoculation. (**a**) The transcript levels of *PmCYP720B11v2* in resistant and susceptible *P. massoniana* after inoculation with PWN. Error bars represent ± SD from three biological repeats. RI, resistant *P. massoniana* inoculated with *B. xylophilus*; RW, resistant *P. massoniana* inoculated with water; SI, susceptible *P. massoniana* inoculated with *B. xylophilus*; SW, susceptible *P. massoniana* inoculated with water. (**b**) The transcript levels of *PmCYP720B11v2* in different tissues of resistant *P. massoniana*. (**c**) Content fold change of diterpenes in resistant *P. massoniana* and susceptible *P. massoniana* after inoculation with PWN at 1, 15, and 30 days. (**d**) Content fold change of diterpenes in resistant *P. massoniana*. Error bars represent ± SD from three biological repeats. Root; Stem; Needle; CK, controls: resistant *P. massoniana* inoculated with water as a control; PWN Inoculation: resistant *P. massoniana* inoculated with PWN over 2 years. Asterisks indicate statistically significant differences from lines expressing *PmCYP720B11v2* genes based on *Student’s t-test*s (*, *p* < 0.05; **, *p* < 0.01).

To further reveal the expression of *PmCYP720B11v2*, qRT–PCR was performed at 1, 15, and 30 days after PWN inoculation using stem tissue from resistant and susceptible *P. massoniana*. Trees inoculated with water were used as controls. The expression of *PmCYP720B11v2* was upregulated in resistant *P. massoniana* compared to the control and was significantly higher than that in susceptible trees at the three sampling points (Figure 2a). It was highly expressed in the roots and stems of resistant *P. massoniana* that grew and developed normally despite containing PWNs for more than 2 years, while a higher expression was not observed in the needles (Figure 2b). Roots are also rich in secondary metabolites and a variety of rhizosphere microorganisms. Pinewood nematodes and rhizosphere microorganisms may induce *PmCYP720B11v2* to be upregulated in the roots. However, the specific reasons are not yet clear and need to be further studied. These results indicated that *PmCYP720B11v2* may participate positively in the defense against the stem-boring PWN.

Since terpene-rich oleoresins are mainly produced in the stem tissue of *P. massoniana*, the higher specific expression of *PmCYP720B11v2* in resistant *P. massoniana* stems suggests that *PmCYP720B11v2* might catalyze diverse terpenoid oxygenation and further improve the terpenoid content, in turn contributing to the defense process of the interaction mechanism between *P. massoniana* and PWN.

**Figure 3:**
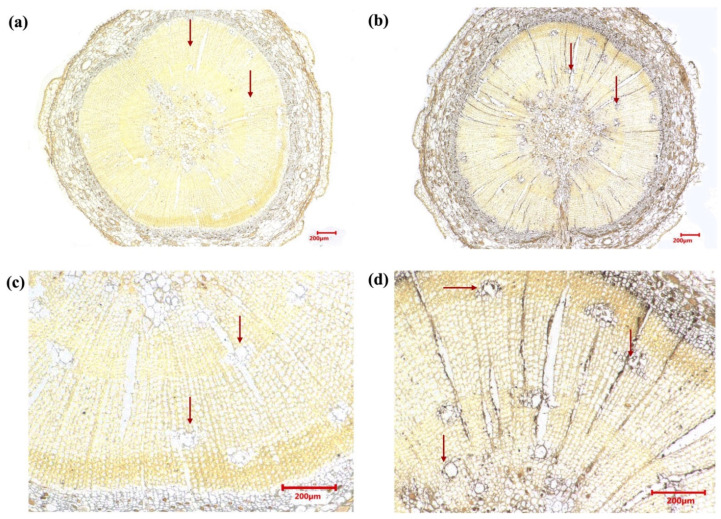
Immunohistochemistry of PmCYP720B11v2 in sections from resistant *P. massoniana*. (**a**) Zoomed view of primary xylem of the stem, cambia, and phloem in sections of the CK. (**b**) Zoomed view of primary xylem of the stem, cambia, and phloem in sections stained for PmCYP720B11v2. (**c**) Zoomed view of resin ducts in sections of the CK. (**d**) Zoomed view of resin ducts in sections stained for PmCYP720B11v2. CK: negative control group without hybridization of specific antibodies to PmCYP720B11v2. Bars, 200 μm.

To further probe the protein-level expression pattern of PmCYP720B11v2, we used immunohistochemistry on sectioned stems of resistant *P. massoniana* to identify the putative proteins. The results indicated that expression signals were not identified in the negative control group without hybridizing PmCYP720B11v2-specific antibodies (Figure 3a,c); in contrast, in the experimental group, a strong expression of a specific signal was visibly identified in the primary xylem of the stem, cambia, and phloem (Figure 3b,d). In particular, the positive signals were most distinct in the epithelial cells of the primary and secondary resin ducts in the xylem (Figure 3d). It can be speculated that the primary xylem of the stem, cambia, and phloem are arranged in abundant resin ducts, where epithelial cells possess multiple glands to secrete and produce oleoresin terpenes [8,31]. P450s combined with diterpene synthases in a modular biosynthetic system can contribute to the considerable structural diversity of conifer DRAs [31], which conforms to the most evident PmCYP720B11v2-specific expression in the epithelial cells of the resin ducts.

**Figure 4:**
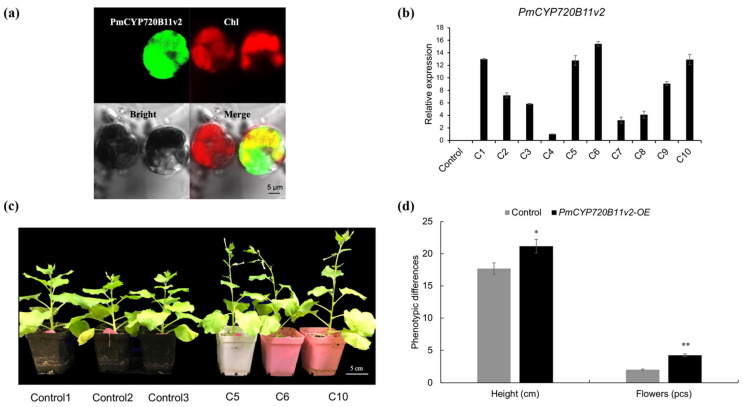
Overexpression of *PmCYP720B11v2* enhanced diterpene production and boosted plant growth and development of transgenic tobacco. (**a**) Subcellular localization of *PmCYP720B11v2.* (**b**) Relative expression of *PmCYP720B11v2* in 10 independent transgenic lines. (**c**) Different phenotypes in positive transgenic tobacco seedlings overexpressing *PmCYP720B11v2*; bar, 5 cm (**d**) Analysis of height and flowers in transgenic tobacco overexpressing *PmCYP720B11v2.* Confidence levels were tested using *Student’s t-tests* (*, *p* < 0.05; **, *p* < 0.01).

For the detection of the subcellular localization of PmCYP720B11v2, green fluorescent protein (GFP) was labeled at the C-terminal end of the gene to verify the subcellular localization pattern by confocal laser scanning microscopy. Based on chlorophyll autofluorescence (Figure 4a), the PmCYP720B11v2-GFP fusion protein appeared in the cytoplasm of protoplasts. This finding suggests that PmCYP720B11v2 is involved in the synthesis of terpenes derived from the cytoplasm.

Furthermore, we also found that the diterpene content in resistant *P. massoniana* stems was significantly increased after PWN inoculation relative to the control (Figure 2c,d). Combined with the transcriptional results of *PmCYP720B11v2*, the immunohistochemical results and the increased diterpene content in resistant *P. massoniana* suggested that *PmCYP720B11v2* might play a positive regulatory role in the defense of resistant *P. massoniana* against PWN.

### 2.3. Overexpression of PmCYP720B11v2 Enhanced Growth and Drought Stress Tolerance in Tobacco

In general, the current studies have been unable to produce a genetic transformation system for *P. massoniana* to further characterize the biochemical function of *PmCYP720B11v2*, and thus, transgenic tobacco lines were produced by the overexpression of *PmCYP720B11v2* under the control of the CaMV 35S promoter (Figure S3). Ten independent transgenic tobacco lines were screened by hygromycin resistance and detected by qRT–PCR (Figure 4b). Three *PmCYP720B11v2* lines (Lines C5, C6, and C10) with higher levels of *PmCYP720B11v2* expression were screened for subsequent characterization.

To further study whether the overexpression of *PmCYP720B11v2* is involved in plant growth and development, phenotypic characteristics were surveyed in *PmCYP720B11v2*-overexpressing lines. Lines C5, C6, and C10 significantly improved the height of the transgenic tobacco and promoted increased flower numbers, indicating that the overexpression of PmCYP720B11v2 efficiently boosted the growth and development of tobacco (Figure 4c,d).

**Figure 5:**
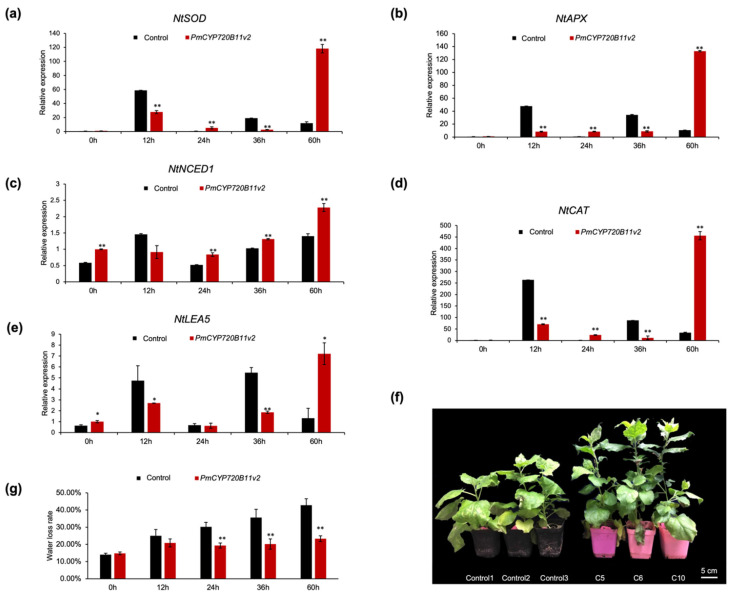
Overexpression of *PmCYP720B11v2* enhanced drought tolerance of transgenic tobacco lines. (**a**) Expression level of *NtSOD* in *PmCYP720B11v2* transgenic tobacco under drought stress. (**b**) Expression level of *NtAPX* in *PmCYP720B11v2* transgenic tobacco under drought stress. (**c**) Expression level of *NtNCED1* in *PmCYP720B11v2* transgenic tobacco under drought stress. (**d**) Expression level of *NtCAT* in *PmCYP720B11v2* transgenic tobacco under drought stress. (**e**) Expression level of *NtLEA5* in *PmCYP720B11v2* transgenic tobacco under drought stress. Data represent the means ± SEs of three biological replicates. Asterisks indicate statistically significant differences from the control. Confidence levels were tested using *Student’s t-tests* (*, *p* < 0.05; **, *p* < 0.01). The 0, 12, 24, 36, and 60 on the x-axis indicate the time of drought treatment (h), and zero represents the control. (**f**) Different phenotypes in positive transgenic tobacco seedlings overexpressing *PmCYP720B11v2* under drought stress; bar, 5 cm. (**g**) Water loss rate of *PmCYP720B11v2*-OE lines and control at 0, 12, 24, 36, and 60 h after drought stress treatment.

To evaluate whether the overexpression of *PmCYP720B11v2* is involved in abiotic attack, a drought stress treatment was performed on Lines C5, C6, and C10. Transgenic tobacco showed drought resistance compared with the control (Figure 5f). Moreover, the expression levels of key genes in response to stress encoding antioxidase (APX, CAD, and SOD), stress-responsive protein (LEA5), and abscisic acid synthase (NtNCED1) were detected. Interestingly, we determined that the expression levels of *NtSOD*, *NtAPX*, *NtCAD*, and *NtLEA5* in *PmCYP720B11v2*-OE lines were lower than the controls before 36 h, which were simultaneously significantly increased at 60 h after drought treatment (Figure 5a,b,d,e). The expression level of *NtNCED1* was significantly higher than that in the controls after 24 h, showing an increasing trend with treatment time (Figure 5c). In order to further determine the drought stress tolerance, we detected the water loss rate between *PmCYP720B11v2* and control. The result showed that the water loss rate of the *PmCYP720B11v2*-OE lines was significantly lower than the control (Figure 5g). Hence, the overexpression of *PmCYP720B11v2* promoted the growth and development of plants and activated the metabolic flux of the antioxidases and stress-responsive proteins to confront adversity.

## 3. Discussion

Epidemics of PWN are causing a serious biotic problem for *P. massoniana* in China and have brought about desperate losses of ecology and economy [8,35]. Conifers have evolved multiple and complicated defense mechanisms to resist many biological and abiological threats throughout history for long-lived survival [10,36,37,38]. Terpenes play integral roles in defense systems against invasive predators or environmental stressors as specific secondary metabolites essential for plants [7,18,39]. *CYP720Bs* are specifically vital enzymes involved in the terpene diversity of conifers that are crucial to understanding the resistance mechanisms [40]. The identification and function of *CYP720Bs* from conifers, especially from resistant *P. massoniana*, are critical and urgent but remain obscure.

### 3.1. Tissue-Specific Expression of PmCYP720B11v2 in P. massoniana in Response to PWN Infection

Plant *CYP450s* are involved in the manufacture of plant hormones, alkaloids, and diterpenoids, contribute significantly to the functional diversity of secondary metabolites in plants, and can be induced by a range of biotic and abiotic stressors. *CYP720Bs* are significant members of the *CYP450* family, specifically in conifers, while the function of the *CYP720Bs* were only characterized in several conifer species, and few *CYP720Bs* genes have been studied in *P. massoniana*. In our study, *PmCYP720B11v2*, a differentially expressed gene in the transcriptomes of resistant and susceptible *P. massoniana*, was first identified. The phylogenetic analysis of the *CYP720B* family from *P. massoniana* in this study showed that *PmCYP720Bs* were divided into four clades (I-IV) (Figure 1a), which is in accordance with the evolutionary characteristics of gymnosperms [10]. *PmCYP720B11v2* was located in clade I of the *PmCYP720B* family, most closely related to *PcCYP720B* from *P. contorta*. Currently, members of the *CYP720B* family have been cloned from a variety of different conifer species, including spruce and loblolly pine. *CYP720Bs* are primarily found in clades I and III. CYP720Bs in clade I can catalyze the direct and efficient conversion of the LAS product 13-hydroxy-8(14)-abietene to 13-hydroxy-8(14)-abietic acid, which can then be hydrolyzed to produce a variety of DRAs without enzymatic catalysis. For example, when *CYP720B2* and *CYP720B12*, members of CYP720B clade I in *P. bankiana*, were tested in vivo, they could catalyze the production of poly (13-hydroxy-8(14)-abietene) DRAs (belonging to diterpenes) directly [30]. *PtCYP720B1* from *P. taeda* is involved in the regulation of DRA production [41,42]. The majority of *PtCYP720B1s* are multifunctional enzymes that can catalyze a range of substrates and synthesize diterpenoids, and further oxidation results in the formation of DRAs [30]. DRAs are critical components of conifer defenses. For instance, they can restrict spore germination, suppress the proliferation of harmful mates of bark beetles, mitigate the tree damage caused by serrated animals, and diminish tall tree tolerance to diseases or pests [32]. We determined the diterpene content in *P. massoniana* stems after PWN inoculation and found that it was significantly increased relative to the control (Figure 2c,d), as was the expression of *PmCYP720B11v2*, which showed *PmCYP720B* may be involved in the biosynthesis of the diterpene response to defend against PWN.

The *CYB720B* family encodes a conifer-specific cytochrome monooxygenase that can be triggered by mechanical injury to the tree or by biotic and abiotic stressors [10,43,44]. At the transcriptional level, in our study we analyzed the expression of *PmCYP720B11v2* in susceptible and resistant *P. massoniana* after PWN inoculation. We found the *PmCYP720B11v2* expressed higher in the resistant *P. massoniana* than the susceptible, and importantly, it was significantly up-regulated in the stems of resistant plants. The stem is the common place where PWN or other weevils land and is also defined as the terpene factory [8]. It can be speculated that *PmCYP720B11v2* positively responds to PWN invasion. *CYP720B19* is involved in the protection of bark tissue against mechanical injury and symbiotic fungal cyanobacteria, the expression of which was considerably increased in the bark tissue of *P. armandi* following 3-4 days of mechanical damage and fungal cyanobacteria and *Heterobasidion annosum* treatment [45,46]. The transcription of defense-related genes (peroxidase, *PaPX2,* and *PaPX3*) was dramatically increased in Norway spruce bark tissue at six days after infection with *H. parviporum* [45]. MeJA therapy can be used to simulate the biotic stressors associated with pests and diseases. After MeJA treatment, the bark expression levels of *CYP720B19* from *P. armandi*; *CYP720B4*, *CYP720B5*, and *CYP720B7* from *Picea asperata*; and *CYP720B1* from *P. taeda* were dramatically enhanced compared to untreated controls [10,29,46]. In Sitka spruce, a transcript analysis revealed tissue-specific expression patterns of *CYP720B2*, *CYP720B12*, and *CYP720B4* in clade I, primarily in stem bark and roots [31]. The expression pattern of *PmCYP720B11v2* in *P. massoniana* following PWN infection was consistent with earlier findings showing that *PmCYP720B11v2* acted as a positive regulator during the process of *P. massoniana* resisting PWN.

At the protein level, we found a stronger expression of PmCYP720B11v2-specific signal was distinctly located in the primary xylem, cambia, and phloem of the stem from resistant *P. massoniana* (Figure 3b,d), which was most distinct in the epithelial cells of the primary and secondary resin ducts in the xylem (Figure 3d). Traumatic resin ducts stimulate the buildup of oleoresin, which provides protection against insects that feed on stems and fungal infections [10]. In spruce species, high concentrations of oleoresin diterpenoids accumulate in the phloem, cambium, wood ray ducts of the xylem, and in the epithelial cells of resin ducts [47,48], which is compatible with the location of PmCYP720B11v2 in *P. massoniana* in our work. Other members of the *CYP720B* family have been found using transcriptome data from loblolly pine and spruce [31], showing that the *CYP720B* family involved in oleoresin production is also part of a larger family in conifer species than just the *TPS* family. In white spruce, CYP720B4 may catalyze the synthesis of 24 distinct diterpenoids belonging to the homologous diterpene, alcohol, aldehyde, and resin acid families [18,31,49]. CYP720B4 is a multisubstrate multifunctional P450 enzyme that contributes significantly to the structural variety of diterpenoid metabolism. Its primary product is abietic acid, which may play a positive role in the defense process for spruce against rhinoceros beetles [32]. These studies were consistent with the increase in diterpene production in resistant *P. massoniana* suffering PWN attack. That is, *PmCYP720B11v2* may participate in a variety of secondary metabolic events that are necessary to understand the chemical composition diversity and flexibility of oleoresin in conifer species and hence contribute to defensive activities.

### 3.2. Overexpression of PmCYP720B11v2 Boosts Growth and Drought Stress Tolerance in Tobacco

The *CYP450* family has a large enzyme protein kingdom in plants. *CYP720Bs* are considered to be a pivotal CYP450 enzymes specific to conifers [19]. They can participate in the synthesis of secondary metabolites, such as terpenoids and plant hormones, and participate in biological and abiotic stresses as a detoxifying substance [31]. To characterize *PmCYP720B11v2*, we observed transgenic tobacco lines inserted with the *PmCYP720B11v2* because the genetic transformation of *P. massoniana* remains currently impossible. We found the height of the transgenic tobacco was significantly improved with increased flowers, which indicated that overexpressed *PmCYP720B11v2* availability contributed to the growth and development in transgenic tobacco (Figure 4c,d). In addition, the phenotypic characteristics of tobacco embedding *PmCYP720B11v2* showed the tolerance to drought stress (Figure 5f), and the loss water rate was lower than the control. The metabolic flux of the antioxidases and stress-responsive proteins were activated. Few overexpression studies of *CYP720Bs* from *pinus* related to abiotic stresses have been performed so far, while whose relatives have been commonly studied in other plants. It is known that plants have evolved complex antioxidant systems, such as SOD, CAT, POD, and stress-responsive proteins to protect cells from oxidative stress when they are damaged by drought stress, which are encoded by the key genes of *APX*, *CAT*, *SOD,* and *LEA5*, respectively [50]. In our study, the transcriptional level of *NtSOD*, *NtAPX*, *NtCAD*, and *NtLEA5* in *PmCYP720B11v2*-OE lines was significantly up-regulated after 60 h under drought stress. In addition, the water loss rate in positive transgenic tobacco seedlings overexpressing *PmCYP720B11v2* under drought stress was significantly lower. In this case, *PmCYP720B11v2* might modulate the expression pattern of these critical genes to protect and stabilize some macromolecules or cellular structures involved in drought stress. In *Arabidopsis*, for instance, *CYP711A1*, a member of the *CYP450* family, is involved in strigolactone biosynthesis and plays a key role in the stress response [51]. Some studies have also revealed that *CYP711A1* positively participated in the flavonoid pathway and influenced shoot branching, which was less responsive to salt treatment in *Arabidopsis* [23,52,53]. Moreover, *CYP450s* play a crucial role in regulating the growth and development of plants [54]. A *CYP450*, *OsDSS1*, is involved in regulating growth and the drought stress response in rice [27]. *CYP89A9* from *Arabidopsis* has been reported to regulate the synthesis of dioxobilin-type catabolites of chlorophyll [55]. In addition, *GmCYP82A3* of soybean enhances stress tolerance based on regulating jasmonic acid and ethylene signal transduction [56]. *MdCYPM1* from apple (*Malus x domestica*) was reported to negatively regulate photomorphogenesis and stress responses in *Arabidopsis* [57]. These studies suggest that *CYP450s* play a key role in plant growth as well as stress responses.

Drought stress, that is, water depletion, has critical negative impacts on plant growth and development, which may be induced by some internal factors of organisms. There is a proposed theory about the pathogenic mechanism of PWN named “tracheid cavitation”. When conifers are infected by PWN, a large amount of volatile substances accumulate inside the pines, and the tracheids are in a hollow state, which hinders the transportation of water in the trees and causes the pine trees to wither [58]. In this work, *PmCYP720B11v2*-overexpressing lines in tobacco showed higher growth and earlier ripening. Furthermore, transgenic tobacco plants showed drought resistance compared with the control. This result indicated that the overexpression of *PmCYP720B11v2* may wake the plant living body and sensitize the specific defense mechanism to cope with hardship. Coupled with the transcriptional and protein expression and tissue location of *PmCYP720B11v2* from resistant *P. massoniana* following PWN inoculation, it may be implicated *PmCYP720B11v2* may positively resist the offensive strategy of PWN in resistant *P. massoniana*.

## 4. Materials and Methods

### 4.1. Nematode Culture

*Botrytis cinerea* was cultured for 5 days on a potato dextrose agar (medium) (PDA) plate in a 28 °C incubator. PWNs were removed from *Pinus massoniana*-carrying PWNs and introduced into the colony, which was cultured at 30 °C until the colony was entirely consumed by the PWNs. The Baermann funnel technique was used to further isolate the PWNs [8,59,60].

### 4.2. Plant Material and Experimental Design

Two-year-old ‘Xiu Ning 5’ (30 individuals) and ‘Huang Shan’ clones (30 individuals) of *Pinus massoniana* in Fuyang (latitude: 30°06′26″ N, 119°59′32″ E), Zhejiang Province of China, were chosen as the materials. They were previously screened according to the PWN-resistance index of *P. massoniana* clones following PWN inoculation, as detailed in Liu et al. [34]. The susceptible clones would wither and die within 30 days after PWN inoculation, so we conducted a long-term phenotype observation and PCR detection of genes from resistant *P. massoniana* individuals after inoculation with PWN. Therefore, in this study, we collected segments from the lateral branches of susceptible and resistant trees at 1 d, 15 d, and 30 d after PWN inoculation. Furthermore, segments of the robust trees still carrying the PWN over 2 years after inoculation were collected, and the water-inoculated trees were taken as the control. Samples of different tissues (root, stem, and needles) were immediately deposited in liquid nitrogen and stored in a freezer at −80 °C for further gene cloning (stem) and real-time quantitative PCR detection (root, stem, and needles). Three biological and technical replicates were performed. Meanwhile, the fresh stem samples were placed in FAA fixative for paraffin embedding and sectioning [8,61]. *Nicotiana benthamiana* was grown in a growth chamber at 26 °C with a 16 h light/8 h dark photoperiod and used for Agrobacterium-mediated transformation [8,60].

### 4.3. Identification of the PmCYP720B Family

PmCYP720B sequences were obtained from our transcriptome (SRP103562) and the full-length transcriptome of *P. massoniana* (PRJNA636925). According to the Pfam database (http://pfam.xfam.org/ (accessed on 4 February 2022)) [62], the domains of the CYP720B proteins in the gymnosperm plant were labeled (Pfam: PF00067). Multiple protein sequence alignments for CYP720Bs of *P. massoniana* were carried out, taking advantage of MAFFT [63], and maximum-likelihood trees were built with CIPRES (https://www.phylo.org (accessed on 4 February 2022)) using the JTT model and 1000 bootstrap replicates [8,60,64].

### 4.4. Gene Cloning and Sequence Analysis

Total RNA was extracted from the stem tissues of resistant *P. massoniana* using an EASY38 Spin Plus Plant RNA Kit (Aidlab, Beijing, China) and was reverse-transcribed to synthesize complementary DNA (cDNA) by a cDNA synthesis kit (Fermentas, Burlington, Ont, Canada). The specific primers of the gene were designed (Table S1) on the basis of the unigene sequences from a reported transcriptome database (SRP103562) and an additional full-length transcriptome (PRJNA636925). To further sequence and identify the full coding sequences, the amplified sequences were ligated to a T-vector PClone007 Blunt Simple Vector Kit (Tsingke, Beijing, China). The full-length CDS of *PmCYP720B11v2* was uploaded into GenBank (Accession No. OM513678) (Figures S1 and S2). The gene was compared to homologous sequences using online BLAST at the National Center for Biotechnology Information (NCBI) (https://blast.ncbi.nlm.nih.gov/Blast.cgi (accessed on 4 February 2022)). The sequence alignment was performed utilizing BioEdit7.2.5 [8,60,65].

### 4.5. Real-Time Quantitative PCR (qRT–PCR) Analysis

Total RNA was extracted and processed as described and reverse-transcribed using a Prime Script RT Reagent Kit (Takara, Dalian, China). Gene-specific primers (Table S1) were designed by Primer Express 3.0.1 (Applied Biosystems, Waltham, MA, USA) for a real-time quantitative PCR (RT–qPCR) analysis of the target genes. RT–qPCR was performed on an ABI PRISM 7300 Real-Time PCR System (Foster City, CA, USA) using SYBR Premix Ex Taq (Takara, Dalian, China). The amplification was conducted in two steps: 95 °C for 30 s followed by 40 cycles at 95 °C for 5 s and 60 °C for 34 s. Three biological replicates were used, and each was checked three times as technical replicates for the qRT–PCR experiments. The data were analyzed using the 2^−ΔΔCt^ method [8,60,66].

### 4.6. Expression and Purification of Recombinant PmCYP720B11v2 Enzyme

The cDNA of *PmCYP720B11v2* was amplified as detailed above, and homologous recombination was used to insert it into pET28a (Invitrogen, Waltham, MA, USA). The resulting recombinant protein was the full-length sequence with a His-tag at the N-terminal end (Figure S3). The expression construct was fully sequence-validated. The plasmid was transformed into competent Rosetta cells (DE3) (Solarbio, Beijing, China).

Five individual colonies were inoculated into Luria–Bertani (LB) medium (3 mL) containing the appropriate antibiotics and incubated overnight at 37 °C and 220 rpm. The LB medium (1000 mL) containing appropriate antibiotics was then inoculated with 1 mL of the overnight culture and grown in a 1000 mL baffle flask at 37 °C and 220 rpm until an optical density (OD) of at least 0.6 at 600 nm was achieved, and then it was cooled to 30 °C. The cultures were induced with 0.5 mM IPTG and then cultured at 30 °C and 220 rpm for approximately 3 h.

The cultures were collected by centrifugation at 8000 rpm for 3 min and resuspended in 50 mL of prechilled NTA-0 buffer (12.11 g Tris base, 146.1 g NaCl, and 500 mL of glycerol dissolved in deionized water were added to 6 mL of concentrated hydrochloric acid to adjust to pH 8.0 and dissolved to 5 L) in an ice bath for 30 min. The ultrasonic parameters set to break the cells were: power 200 W, work 3 s and pause 4 s for 99 cycles. The cell pellets were clarified by centrifugation (50 min, 16,000× *g*, 4 °C). The cleared lysates were filtered with a 0.22 µm filter, applied to a His SpinTrap Ni-affinity column (GE Healthcare, Piscataway, NJ, USA), and eluted with 20 mM, 60 mM, 200 mM, and 500 mM imidazole, pH 8.0, at 4 °C following Coomassie brilliant blue G250 detection.

After SDS–PAGE electrophoresis, the proteins were transferred to polyvinylidene difluoride (PVDF) membranes by wet transfer at 100 V for 1–2 h, and then the membrane was washed three times with PBST buffer for 10 min each time. The membranes were placed into PBST containing 5% nonfat milk powder for blocking at 37 °C for 1 h and washed three times with PBST buffer. The membranes were placed into a 1:5000 dilution of anti-His antibody, incubated for 1 h at 37 °C, and washed three times with PBST buffer. An ECL chemiluminescence detection kit was used for color development. Equal volumes of liquid A and liquid B were mixed and applied to the PVDF membrane for 1 min, and then the results were observed in the dark with X-ray exposure for 2 min [8,60].

### 4.7. Polyclonal Antibody Preparation

The pET28a prokaryotic expression vector *PmCYP720B11v2* was constructed, and the encoded proteins were successfully expressed, purified, and then identified by Western blotting (Figure S4). The recombinant protein was used as an antigen to produce specific antibodies. Two Japanese rabbits were immunized with a protocol of 1 mg/0.5 mg/0.5 mg. ELISA was performed to detect the antibody potency using an immunogen-coated ELISA plate at a concentration of 1 µg mL^−1^ and secondary antibody (HRP-conjugated goat anti-rabbit IgG) at concentrations of 1:10,000 and 450 nm to meet the requirements of the antiserum ELISA. Titers were as low as 1:50,000. A final antiserum dilution of 1:64,000 was detected, and an antigen OD greater than 1 was detected [8,60].

### 4.8. Immunohistochemistry

To further investigate the expression pattern of PmCYP720B11v2, we performed immunohistochemistry. Stems of resistant *P. massoniana* were cut into 1 mm slices and fixed in formalin, and then the stem tissues were embedded, sectioned, and stained according to the method of Chang et al. [61]. The embedded tissues were cut into 5 µm cross-sections by a microtome (Leica, Weztlar, Germany) and subjected to a dewaxing process according to the method of Chen et al. [67], followed by two washes in 0.1 M PBS buffer (pH 7.4) for 5 min each to prepare for further immunohistochemistry [68]. Sections were blocked with immunostaining blocking solution at 25 °C for 15 min and then hybridized overnight at 4 °C with a 1:200 dilution of anti-PmCYP720B11v2 primary specific polyclonal antibody. Sections were washed three times in PBS buffer for 10 min each and incubated with an HRP-labeled goat anti-rabbit secondary antibody at a 1:50 dilution for 2 h at 25 °C. After the last PBS wash, the reaction was observed with a DAB horseradish peroxidase color development kit for 15 min, followed by observation with a VHX-5000 super deep scene microscope until the sections were air-dry protected from the light [8,60].

### 4.9. Subcellular Localization of the PmCYP720B11v2 Gene

*PmCYP720B11v2* binds to the C-terminus of green fluorescent protein (GFP) to produce a fusion protein under the control of two 35S promoters (Figure S3). The primers used for sequence amplification are shown in Table S1. Protoplasts from tobacco leaves were isolated, plasmid DNA was introduced by a polyethylene glycol (PEG)-mediated transformation as detailed previously [69], and then the protoplasts were incubated at 28 °C for 16 h in the dark. For the visualization of GFP, the excitation wavelength was 488 nm, and emitted fluorescence was collected after passing through a broadband pass filter (450–550 nm) taking advantage of a ZEISS LSM-700 confocal microscope [8,60,70].

### 4.10. Generation of PmCYP720B11v2 Transgenic Lines in Tobacco

To construct the overexpression vector of *PmCYP720B11v2*, a full-length CDS of a 1461 bp fragment was amplified and inserted into the pCambia1300S vector. The primers for PCR amplification are shown in Table S1. Leaf discs of *N. benthamiana* were transformed by EHA105 of the *A. tumefaciens* strain containing the pCambia1300S-*PmCYP720B11v2* construct as described previously, as was the control construct pCambia1300S vector [8,60]. Ten independent *PmCYP720B11v2* transformed lines were obtained. The transformed plants obtained after selection with hygromycin were confirmed by genomic DNA PCR using gene-specific primers (Table S1). qRT–PCR was performed as detailed above, and actin was used as the reference gene.

### 4.11. Oleoresin Terpene Extraction from P. massoniana

Oleoresin terpenes were collected from the stems of resistant *P. massoniana* after PWN inoculation over 2 years. Stem materials were collected and chopped with a pruning shear, and 1 g was dissolved in 0.5 mL of ethanol for 12 h to extract the terpenes. The content of the oleoresin terpene chemical components in *P. massoniana* was determined by GC–MS, as detailed by Karanikas et al. [8,60,71].

### 4.12. GC–MS Analysis

The chemical composition of oleoresin terpenes in *P. massoniana* was determined by GC–MS with an Agilent 7890 gas chromatograph (Agilent J&W Scientific, Santa Clara, CA, USA) equipped with an Agilent 5975 mass spectrometer. Chemicals were extracted using an SPME fiber coated with 50/30 μm divinylbenzene/carboxen/polydimethylsiloxane (DVB/CAR/PDMS) (Supelco Co., Bellefonte, PA, USA). Ground samples were incubated for 30 min at 40 °C, followed by incubation with polydimethylsiloxane (19,091 S-433) (60 m × 0.25 mm internal diameter, 0.25 μm film thickness) on a fused silica capillary column (DB-5MS) for the differentiation of volatiles. Helium was used as the carrier gas at a concentration of 1.0 mL min^−1^. The column oven program temperature was started at 50 °C (hold for 2 min), then ramped to 80 °C at 3 °C min^−1^ (hold for 2 min), followed by a second ramp to 180 °C at 5 °C min^−1^ (hold for 1 min), a third ramp to 230 °C at 10 °C min^−1^ (hold for 1 min), and then a final ramp to 250 °C at 20 °C min^−1^ (hold for 3 min). The MS conditions were as follows: ion source, 230 °C; electron energy, 70 eV; GC–MS interface zone, 250 °C; and a scan range of 50–500 mass units. Ethyl decanoate was used as an internal standard and was further validated by comparing the electron ionization mass spectra with those in the NIST Mass Spectral Library. The analysis of the terpene chemical content of *P. massoniana* was performed using isobutylbenzene as the monoterpene and sesquiterpene internal standard and heptadecanoic acid as the diterpene internal standard. Three biological and three technical replicates were performed [8,60].

### 4.13. Drought Stress Treatment

Six-week-old wild-type and *PmCYP720B11v2*-overexpressing tobacco plants were subjected to 20% PEG to simulate drought stress, and phenotypic characteristics were surveyed and water loss rate was detected after 0, 12, 24, 36, and 60 h, referring to Duan et al. (2017). qRT–PCR was used to determine the expression changes of stress-related genes in wild-type and transgenic tobacco before and after drought stress. The gene and primer sequences are shown in Table S1 [28].

## 5. Conclusions

In conclusion, we characterized the function of *PmCYP720B11v2* in resistant *P. massoniana* after PWN inoculation. The *PmCYP720B11v2*-specific expression pattern and its localization demonstrated that it could have a favorable role in the metabolic and defensive processes of oleoresin diterpenes in response to a stem-boring PWN attack. In addition, the overexpression of *PmCYP720B11v2* boosted plant growth dominance and activated the metabolic flux of antioxidases and stress-responsive proteins under drought conditions, providing a novel metabolic engineering scenario to enhance the growth and drought stress tolerance of tobacco.

## Data Availability

All data generated or analyzed during this study are included in this article (and its Supplementary Materials). The gene sequence raw data were uploaded into GenBank (https://www.ncbi.nlm.nih.gov/WebSub/ (accessed on 4 February 2022)) (Accession No. OM513678).

## Supplementary Materials

The following supporting information can be downloaded at: https://www.mdpi.com/article/10.3390/ijms23126640/s1.
