# Supplementary material for: Identification and Defensive Characterization of PmCYP720B11v2 from Pinus massoniana
Source: Int J Mol Sci. 2022 Jun 14;23(12):6640. doi: 10.3390/ijms23126640 (PMC9223603; doi:10.3390/ijms23126640)
Supplement: Supplementary file 1 [file ijms-23-06640-s001.zip › ijms-1711862-supplementary.pdf]

The following Supporting Information is available for this article:

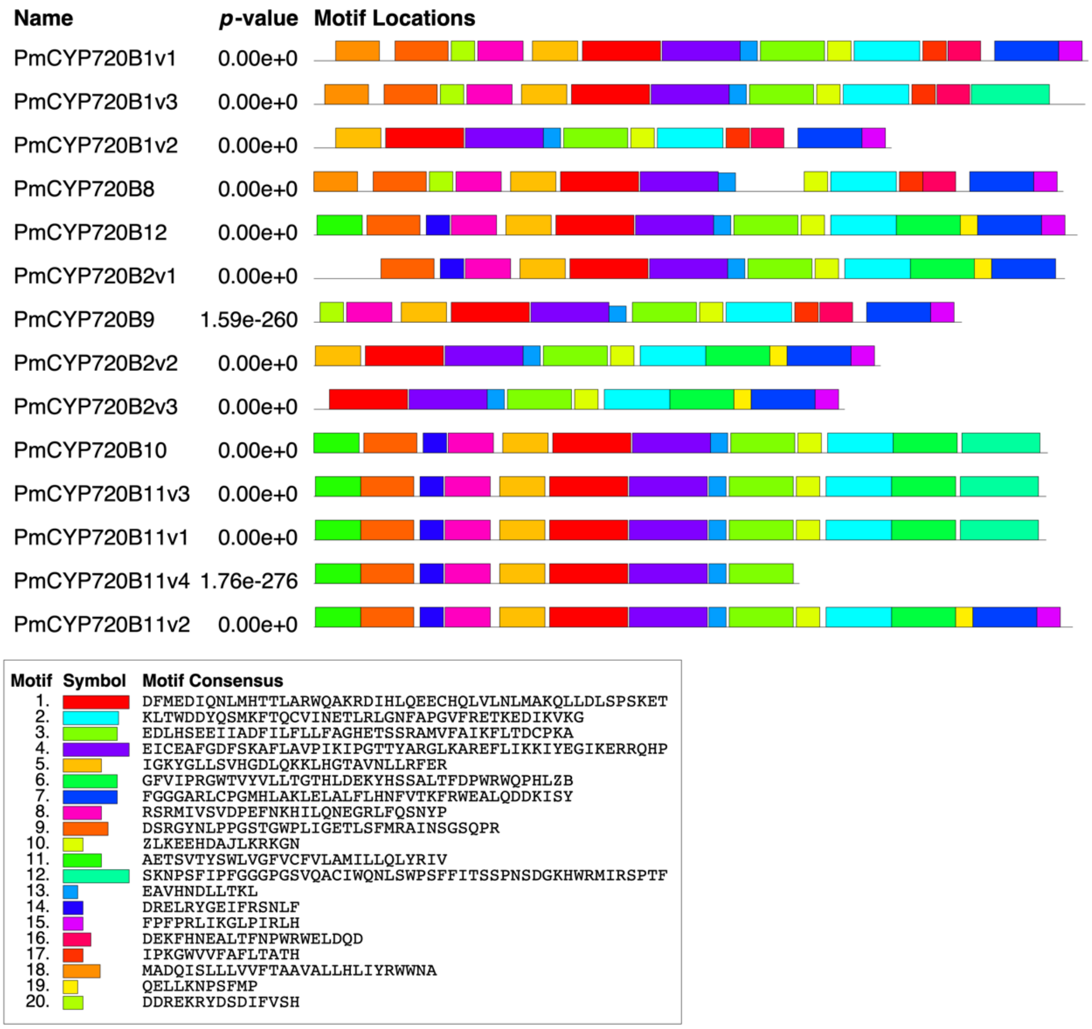

Figure S1. Conserved motif analysis of the PmCYP720Bs in *P. massoniana.*

>PmCYP720B11v2

ATGGCGGAGACTAGTGTTACGTATTCATGGCTGGTGGGCTTCGTCTGTTTCGTTCTGGCGATGATACTG
CTTCAGCTCTACAGAATAGTGGACAGTCGAGGCTACAATTTACCTCCCGGTTCCAGAGGGTGGCCATTG
ATTGGAGAATCCTTGGGCTTCATGCGAGCGATTAATTCGGGTTCACAACCACGCCGATTCATTCAAGAT
CGAGAGCTAAGGTATGGCGAGATATTCAGATCAAATTTGTTCGGAAGATCTCGAATGATTGTGTCTGTG
GATCCAGAATTCAACAAACATATTCTGCAAAATGAAGGCAGGCTGTTTCAATCCAATTATCCGAGACCT
TTTCGAAATCTCATCGGCAAATTCGCTTTGCTTTCGGTGCACGGAGATCTCCAGAAGAAGCTCCACGGA
ACGGCCGTAAATTTCCTGGGGTTCGAGAAACTGCGTGTGCACTTCATGGACGACATACAGAACCTTATG
CAAACCACCTTCGCCCAGTGGCAAGCCAAGAGGGATATCCATCTTCACGAGGAGTGCCATCAGCTTGTT
CTGAATTTGATGGCAAAACAATTGCTGGACTTATCTCCGTCCAAGGAGACAGAAGAGATCGGTAAGGCA
TTCGCCGCTTTTTCTAAAGCTTTCCTTGCCGTTCCCATTAGAATCCCGGGTACCTCGTACGCCAGAGGA
ATTAAGGGCAGAGAGTTTCTGATAAAAACGATTAATGAGGGTATAAAGCACAGAAGGCAGCATCCAGAT
GAAGCTGTCCACAATGATTTCTTGGGAGAACTCTTGAAAGAAGACTTGCATTCAGAAGAAGTTATAGCA
GATTTAGTATTATTCCTGCTCTTCGCTGGTCACGAGACCTCAGCCTCAGCGATGGTATTTTCTATCAAA
TTTCTCACGGACTGTCCCCAGGCACTTCACGAACTGAAGGAGGAGCACGGCGCTCTGTTAAAGAGAAAG
GGGAGTCCAAGAAACCAAAAGCTCACTTGGGATGACTACCAGTCAATGAAATTCACCCAATGTGTCATA
TATGAAACACTTCGTCTCGCCAACGTCGCTTCAGCGCTTTACAGAGAAGCAAAAGAAGACATTAAAATT
AAAGGAGGCTTTGTGATTCCAAGAGGGTGGACAGTCATGATACTTCTGAACAGCATGCATCTGGACGAA
AAGTACCATTCTTCTGCGCTCACATTTGACCCATGGCGTTGGCAACGGCAGCTCGAAAATAATGAGCTC
TCAAAGAACCCCTCTTTTATACCTTTTGGAGGGGGGGCCAGGCTCTGTCCAGGCATGCATCTGGCAAAA
CTTGAGCTGGCCCTCTTTCTTCATAACTTCGTCACCAAATTCAGATGGGAAGCACTGGAGGATGATAAG
ATCTCCTACTTTCCTGTTTCTCACTTGACTAAAGGCTTTCCGATCCGTCTACATCATCTTCAAGAACGA
ATGGACGATTAA

>PmCYP720B11v2

MAETSVTYSWLVGFVCFVLAMILLQLYRIVDSRGYNLPPGSRGWPLIGESLGFMRAINSGSQPRRFIQD
RELRYGEIFRSNLFGRSRMIVSVDPEFNKHILQNEGRLFQSNYPRPFRNLIGKFALLSVHGDLQKKLHG
TAVNFLGFEKLRVHFMDDIQNLMQTTFAQWQAKRDIHLHEECHQLVLNLMAKQLLDLSPSKETEEIGKA
FAAFSKAFLAVPIRIPGTSYARGIKGREFLIKTINEGIKHRRQHPDEAVHNDFLGELLKEDLHSEEVIA
DLVLFLLFAGHETSASAMVFSIKFLTDCPQALHELKEEHGALLKRKGSPRNQKLTWDDYQSMKFTQCVI
YETLRLANVASALYREAKEDIKIKGGFVIPRGWTVMILLNSMHLDEKYHSSALTFDPWRWQRQLENNEL
SKNPSFIPFGGGARLCPGMHLAKLELALFLHNFVTKFRWEALEDDKISYFPVSHLTKGFPIRLHHLQER
MDD

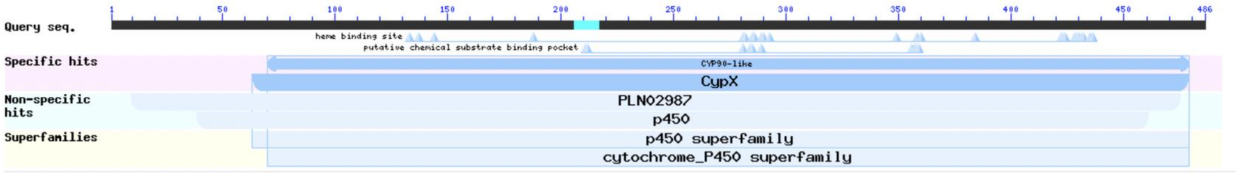

Figure S2. Sequences of cDNA, amino acid and the domain information of *PmCYP720B11v2* from *P. massoniana.*

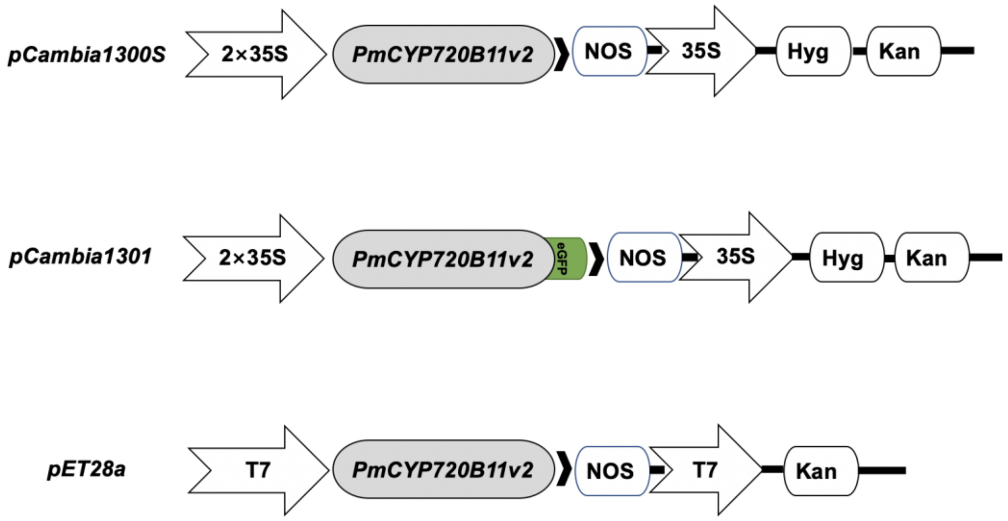

Figure S3. Vectors for *PmCYP720B11v2* from *P. massoniana*. 35S, CaMV 35S promoter; T7, T7 promoter. NOS: Nopaline synthase terminator; Hyg, Hygromycin resistance gene; Kan, Kanamycin resistance gene.

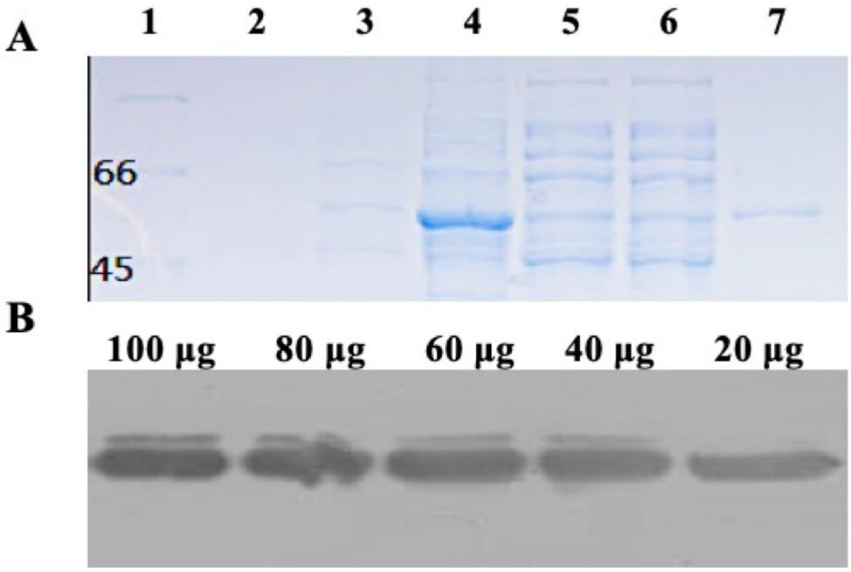

Figure S4. Purification and Western blot of PmCYP720B11v2. A. Lane 1, protein marker: serial bands. Lane 2, Pre-inducible protein; Lane 3, After-inducible protein; Lane 4, Bacterial precipitation; Lane 5, Protein effluent. Lane 6, Wash protein; Lane 7, Purified Protein; B. Western blot verification of PmCYP720B11v2.

Table S1. Primers used in the paper.

Primers name	Primers sequences (5'-3')
PmCYP720B11v2-F	ATGGCGGAGACTAGTGTTAC
PmCYP720B11v2-R	ATCGTCCANTTCGTTCTTG
PmCYP720B11v2-VF	tcagcagtcgaagagcATGGCGGAGACTAGTGTTAC
PmCYP720B11v2-VR	ttagcgtgtgaagagcATCGTCCANTTCGTTCTTG
PmCYP720B11v2-qF	TGGCGATGATACTGCTTCAG
PmCYP720B11v2-qR	AAGCCCAAGGATTCTCCAAT
PmActin4-F	TGCCTGC CATGTATGTTGCA
PmActin4-R	GTCAGAT CACGCCCAGCAAG
NtAPX-F	CAAATGTAAGAGGAAACTCAGAGGA
NtAPX-R	AGCAACAACTCCAGCTAATTGATAG
NtCAT-F	AGGTACCGCTCATTCACACC
NtCAT-R	AAGCAAGCTTTTGACCCAGA
NtSOD-F	CCGTCGCCAAATTGCATAG
NtSOD-R	CGATAGCCCAACCAAGAGAAC
NtNCED1-F	CTATTTCCACTTCAAAACCAACCAC
NtNCED1-R	GGCACTTTCCACGGCATCT
NtLEA5-F	GAACCCAACAAGAGCGAGAGA
NtLEA5-R	CGACAGGAAGCATTGACGAG
Actin-F	TCCTGATGGGCAAGTGATTAC
Actin-R	GAATCCACGAGACCACATACAA
